# Stop-Flow Lithography for the Continuous Production of Degradable Hydrogel Achiral Crescent Microswimmers

**DOI:** 10.3390/mi13050798

**Published:** 2022-05-20

**Authors:** Junfeng Xiong, Xiaoxia Song, Yuhang Cai, Jiahe Liu, Yangyuan Li, Yaqiang Ji, Liang Guo, U Kei Cheang

**Affiliations:** 1 School of Mechatronics Engineering, Harbin Institute of Technology, Harbin 150001, China; 11849603@mail.sustech.edu.cn (J.X.); 11849530@mail.sustech.edu.cn (Y.J.); 2 Department of Mechanical and Energy Engineering, Southern University of Science and Technology, Shenzhen 518055, China; 11930824@mail.sustech.edu.cn (X.S.); 12132244@mail.sustech.edu.cn (Y.C.); jiaheliu2-c@my.cityu.edu.hk (J.L.); 11711207@mail.sustech.edu.cn (Y.L.); guol3@sustech.edu.cn (L.G.); 3 Shenzhen Key Laboratory of Biomimetic Robotics and Intelligent Systems, Southern University of Science and Technology, Shenzhen 518055, China; 4 Guangdong Provincial Key Laboratory of Human-Augmentation and Rehabilitation Robotics in Universities, Southern University of Science and Technology, Shenzhen 518055, China

**Keywords:** stop-flow lithography, degradable hydrogel microswimmers, crescent, magnetic actuation

## Abstract

The small size of robotic microswimmers makes them suitable for performing biomedical tasks in tiny, enclosed spaces. Considering the effects of potentially long-term retention of microswimmers in biological tissues and the environment, the degradability of microswimmers has become one of the pressing issues in this field. While degradable hydrogel was successfully used to prepare microswimmers in previous reports, most hydrogel microswimmers could only be fabricated using two-photon polymerization (TPP) due to their 3D structures, resulting in costly robotic microswimmers solution. This limits the potential of hydrogel microswimmers to be used in applications where a large number of microswimmers are needed. Here, we proposed a new type of preparation method for degradable hydrogel achiral crescent microswimmers using a custom-built stop-flow lithography (SFL) setup. The degradability of the hydrogel crescent microswimmers was quantitatively analyzed, and the degradation rate in sodium hydroxide solution (NaOH) of different concentrations was investigated. Cytotoxicity assays showed the hydrogel crescent microswimmers had good biocompatibility. The hydrogel crescent microswimmers were magnetically actuated using a 3D Helmholtz coil system and were able to obtain a swimming efficiency on par with previously reported microswimmers. The results herein demonstrated the potential for the degradable hydrogel achiral microswimmers to become a candidate for microscale applications.

## 1. Introduction

Microswimmers refer to controllable swimming microrobots that are micron-sized and can be controlled to perform tasks with high precision. The advantage of small size gives them enormous potential in many applications such as minimally invasive surgery, environment monitoring, and cell transportation [1]. Many different types of microswimmers are actuated in different ways, such as chemical, magnetic, light, and electric field actuation [2,3,4,5]. In particular, magnetic actuated microswimmers are one of the most promising microswimmers because the magnetic field can penetrate most materials, and it allows precise control of the microswimmers over a long range [6]. Over the years, researchers have extensively studied various types of magnetic microswimmers that can swim in low Reynolds number environments [7,8,9], such as helical, flexible, and 2D achiral microswimmers. In recent years, a heavier emphasis was placed on the practical applications of these microswimmers; thus, improving the degradability of microrobots to reduce the physical legacy impact of the microswimmers after finishing biomedical or environmental monitoring tasks has become one of the most pressing issues. Many degradable hydrogels have been successfully used to prepare microswimmers, such as poly (ethylene glycol) diacrylate (PEGDA), gelatin methacryloyl (GelMA), and poly (lactide-co-glycolic acid) (PLGA) [10,11,12,13,14,15]. Peters et al. fabricated the helical hydrogel microswimmers for theranostic cargo delivery using two-photon polymerization [16]. This work demonstrated the concept of combining the helical microswimmer design with degradable PEGDA hydrogel nanocomposite. Enzymatically biodegradable microswimmers were reported as follow-up studies demonstrating controlled biodegradability in collagenases of different concentrations by using GelMA hydrogel as the fabrication material [6,17]. The magnetic nanoparticle retrieval technique was also introduced to demonstrate the concept of removing excess particles after the bodies of the hydrogel microswimmers were fully degraded in NaOH [12]. However, most reported hydrogel microswimmers could only be fabricated using two-photon polymerization (TPP) due to their 3D structures [6,16,17]. The print speed of 5–10 s for each microswimmer may limit the preparation efficiency of the degradable microswimmers and increase the cost of use, thus limiting the potential of hydrogel microswimmers to be used in applications where a large number of microswimmers are needed.

Here, we proposed a new type of fabrication method for degradable hydrogel microswimmers using a custom-built SFL setup. SFL is a semi-automatic processing technology, and it was widely used in mass manufacturing multifunctional microstructures [18,19,20]. Sharan et al. fabricated catalase-propelled hydrogel micromotor via SFL [21], demonstrating the feasibility of massively manufacturing 2D shapes using SFL. The hydrogel microswimmers in this work feature a 2D crescent design and are composed of magnetic PEGDA nanocomposite. The shape design and the choice of material allow for the mass fabrication of magnetically driven microswimmers with controlled degradability. The magnetic PEGDA nanocomposite is an artificial hydrogel material with good biocompatibility, superparamagnetism, and degradability [22]. The components of the hydrogel nanocomposite include cross-linkable monomer PEGDA, photoinitiator, and Fe_3_O_4_ magnetic nanoparticles. Using a photomask with crescent patterns and hydrogel nanocomposite, we were able to mass fabricate hydrogel crescent microswimmers. In the degradability test, the hydrogel achiral crescent microswimmers were fully degradable in less than 70 min through accelerated hydrolysis. The degradation rate of the hydrogel microswimmers was controlled by adjusting the concentration of NaOH. Cytotoxicity assays showed that the cytotoxicities of the hydrogel precursor and the hydrogel crescent microswimmers were relatively low. In addition, the movement ability of the crescent microswimmers was also tested. When actuated with an external rotating magnetic field (RMF), the microswimmers were able to swim forward by converting rotational motion into translational motion. These experiments showed that the hydrogel crescent microswimmers prepared by SFL have good degradability and swimming efficiency, providing a new option for fabricating hydrogel robotic microswimmers.

## 2. Materials and Methods

### 2.1. Preparation of Magnetic Hydrogel Nanocomposite

The hydrogel achiral crescent microswimmers were formed by the polymerization of hydrogel nanocomposite through UV light. The hydrogel nanocomposite was composed of PEGDA (Sigma-Aldrich), photoinitiator (2-hydroxy-2-methyl-1-phenyl-1-propanone, Darocur 1173, Sigma-Aldrich), and water-based ferrofluid containing Fe_3_O_4_ nanoparticles with 10 nm hydrodynamic size (EMG 700 SP, Ferrotec, Bedford, OH, USA). The hydrogel precursor (30 vol% PEGDA, 3 vol% Darocur 1173, and 67 vol% water) was treated with ultrasonic oscillation (SB-5200D, Ningbo Xinyi, Ningbo, China) for 10 min (The crosslinking yield of PEGDA hydrogel is 33.5% and the swelling efficiency is 100.3%. The data are shown in Table S1). An appropriate amount of water-based ferrofluid (the final mass fraction of Fe_3_O_4_ is 5 wt.%) was added, and the mixture was under ultrasonic oscillation for 30 min to obtain a uniformly dispersed mixture. Tween-20 (0.05 vol%, Sigma Aldrich, St. Louis, MO, USA) was used to prevent microswimmer loss due to sticking on tubes. Deionized (DI) water was used in all experiments. CCK-8 kit and Calcein-AM/PI Double Stain Kit (C2015S) were obtained from Dojindo (Kyushu, Japan) and Biyuntian Biotechnology (Shanghai, China), respectively.

### 2.2. Fabrication of Microfluidic Chips

The hydrogel nanocomposite was injected into the microchannel of the microfluidic chip using a syringe pump. The hydrogel crescent microswimmers were fabricated from UV-curing the hydrogel nanocomposite. The microfluidic chip was fabricated using soft lithography [23]. First, an SU-8 (Microchem) positive template was prepared with standard photolithography technology. Second, polydimethylsiloxane (PDMS, Sylgard 184, Dow Corning) was poured into the SU-8 positive template, evenly mixed, and kept in a vacuum environment for 10 min to remove bubbles in the PDMS. Then, the positive template with PDMS was placed in an oven at 80 °C for 1 h. Next, the molded PDMS channel was carefully torn off from the edge and cut into a size of 4 cm × 1 cm × 7 mm. Inlet and outlet holes were created on this PDMS channel using a hole puncher. Afterward, a glass slide was covered with a 100 μm thick PDMS layer. Finally, the PDMS channel was bonded to the PDMS-coated glass slide by plasma (Harrick Plasma, New York, NY, USA.). After the bonding, the PDMS chip was kept in an oven at 60 °C for 30 min to obtain a stronger bonding effect. The dimensions of the PDMS microchannel in the microfluidic chip were 40 μm high, 1100 μm wide, and 3 cm long.

### 2.3. Stop-Flow-Lithography Setup

**Figure 1:**
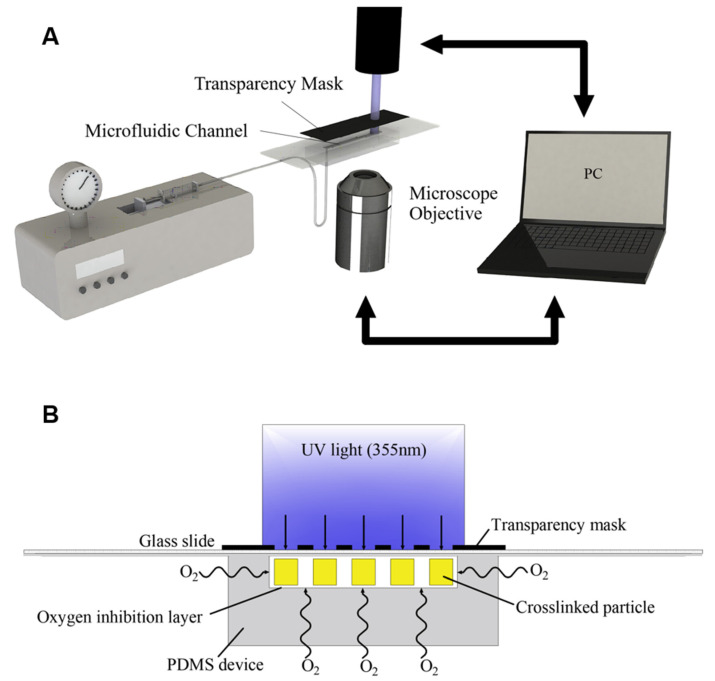
(**A**) Schematic diagram of SFL. (**B**) The hydrogel nanocomposite in the microchannel undergoes cross-linking polymerization under ultraviolet (UV) light.

A custom-built SFL setup was used for the fabrication of hydrogel achiral crescent microswimmers (the setup is shown in Figure S1). The setup consisted of a microfluidic device and a laser system. The schematic diagram of the system is shown in Figure 1A. The laser system included an ultraviolet light excitation system and a visible light imaging system. The ultraviolet light was generated by a 355 nm laser source (Talon-355-15NL, Spectra-Physics, Tokyo, Japan), and a collimated light beam was formed through an attenuator and a beam expander, the spot diameter of which is 2 cm. The visible light imaging system included a CCD camera, an LED light source, and an objective lens (5× or 10×), which was used to observe the synthesis of the hydrogel microswimmers in the microchannel. The microfluidic device consisted of five parts: a syringe pump, a 2 mL syringe, a microfluidic chip, a photomask, and a collection tube, which were connected by soft catheters.

### 2.4. Fabrication of Hydrogel Crescent Microswimmers

SFL can perform flow–stop polymerization in cycles to achieve continuous production of the hydrogel crescent microswimmers. In the flow stage, the microchannel was filled with the hydrogel precursor suspension through the micropump. In the stop stage, the micropump was turned off, and the hydrogel precursor flow stops in a short time. In the polymerization stage, the 355 nm laser source was turned on to emit ultraviolet light, which was projected into the microchannel through a patterned photomask. Under UV exposure (light intensity around 152 mW/cm^2^ based on the average power, exposure time 4 s, repetition rate 5 kHz), the hydrogel precursor was quickly cross-linked and solidified to form the hydrogel crescent microswimmers. The effective projection area of UV light is a circle of around 2 cm diameter, which enables the polymerization of around 400 microswimmers in the channel within one cycle. Complete fabrication cycles were successfully run in 24 s (*t*_flow_ = 5 s; *t*_stop_ = 15 s; *t*_pol_ = 4 s). Note that the oxygen that penetrated the microfluidic chip inhibited the cross-linking polymerization reactions; as a result, a 2–3 μm thick lubricating layer was formed around the microchannel, where no crosslinking occurred. This layer is known as an oxygen inhibition layer (Figure 1B) [24]. The oxygen inhibition layer allowed the solidified hydrogel crescent microswimmers to be easily flushed out by pressure-driven flow without sticking to the surface of the microchannel. The preparation of the hydrogel crescent microswimmers can be performed continuously by repeating the flow-stop-polymerize cycle. The flushed-out hydrogel crescent microswimmers were collected into the collection tube. Isopropyl alcohol was added to dissolve the excess hydrogel precursor. Centrifugation (5000 RPM, 5 min) was used to precipitate the hydrogel microswimmers, and the supernatant was removed. Finally, the hydrogel microswimmers were washed 5 times with 0.05 vol% Tween 20 solution.

### 2.5. Structural Characterization

The surface morphology and the elemental composition of the hydrogel crescent microswimmers were characterized using scanning electron microscopy (SEM, ZEISS, Merlin, OR, USA) at 5 keV and 1 nA and energy-dispersive spectrometry (EDS, EDAX, Mahwah, NJ, USA, Octane Pro), respectively.

### 2.6. Degradation Test

The degradation behavior of the hydrogel crescent microswimmers was quantitatively analyzed by observing their degradation rate under different NaOH concentrations [12,16]. Different hydrogel crescent microswimmers were immersed in NaOH solutions with concentrations of 1 mol/L, 3 mol/L, and 5 mol/L, respectively. The samples were sealed and stored at 25 °C. An optical microscope (Mingmei, Guangzhou, China) was used to obtain brightfield images of the microswimmers every 10 min. The pixel statistics function of the camera was used to mark the surface area of the microswimmer during degradation.

### 2.7. Cell Culture

The L929 cells were cultured in MEM complete medium, which included 90% glucose-free MEM (GIBCO, California, USA), 10 vol% fetal bovine serum (FBS, GIBCO), and 1 vol% penicillin/chain Mycin (10,000 U/mL, GIBCO). The cells were maintained at 37 °C in humidified air with 5 vol% CO_2_.

### 2.8. Cell Viability

The CCK-8 cell viability kit was used to quantitatively determine the cytotoxicity of the samples. The mouse fibroblast cell line L929 was seeded in 96-well microplates with 100 μL of MEM complete medium/well at a concentration of 1 × 10^5^ cells and stabilized in an incubator for 12 h. Then, different samples were added to 96-well tissue culture plates. After culturing for 24 h, 10% CCK-8 solution was added to the cell culture. The cell culture was incubated for another 1 h at 37 °C. The absorbance of the samples was measured at 450 nm using a multifunctional full-wavelength micro-plate reader (Infinite 200 pro, Tecan Austria GmbH, Grodig, Austria). Calcein acetoxymethyl ester(calcein-AM) and propidium iodide (PI) double staining kits were used for cell live/dead staining. The cells were seeded in 96-well cell culture plates at a density of 1 × 10^5^ cells per well. Then, different samples of 50 uL with the concentration of 2k microswimmers/mL were added to the corresponding wells. the microswimmers were in full contact with the cells. After 24 h of incubation, the cells were washed twice with PBS. Then, 100 μL Calcein-AM/PI double stain detection working solution was added according to the manufacturer’s instructions. After incubating for 30 min, the cells were rinsed twice with PBS. Finally, a confocal microscope (A1R+Symp64, Nikon, Tokyo, Japan) was used to observe Calcein-AM-stained live cells (λ_excitation_/λ_emission_ = 490 nm/515 nm) and PI-stained dead cells (λ_excitation_/λ_emission_ = 535 nm/617 nm).

### 2.9. Motion Control Tests

The hydrogel crescent microswimmers were actuated using a magnetic control system composed of an imaging system, which consisted of a microscope and a CMOS camera, and a three-dimensional Helmholtz coil system [17,25]. The solution containing the hydrogel microswimmers was transferred to a petri dish via pipetting. A 10 mT RMF was used to manipulate the microswimmers. By adjusting the rotating frequency and direction of the rotating magnetic field, the swimming velocity and direction of the microswimmers can be tuned. The petri dish containing microswimmers was placed on the platform at the center of the magnetic control system to get a uniform field. The imaging system recorded the motion of the microswimmers at a frame rate of 25 Hz. An image processing algorithm written with MATLAB was used to analyze the velocities and trajectories of the microswimmers.

## 3. Results

### 3.1. Characterization of Hydrogel Crescent Microswimmers

**Figure 2:**
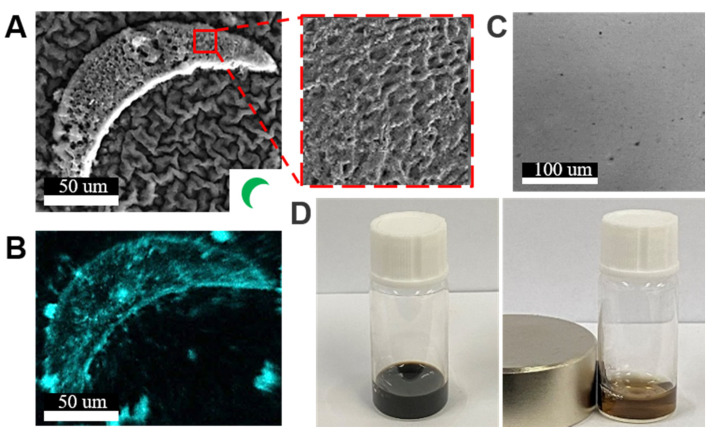
(**A**) SEM image of a hydrogel crescent microswimmer. The inset figure in the lower right corner is the pattern on the transparent mask used to make the microswimmers. The zoomed-in figure shows the porous structures of the microswimmer. (**B**) The EDS of the iron element indicated the presence of Fe_3_O_4_. (**C**) Optical image of Fe_3_O_4_ nanoparticles uniformly dispersed in the hydrogel precursor suspension. (**D**) When the neodymium iron boron magnet was placed close to the hydrogel precursor suspension, the Fe_3_O_4_ nanoparticles gathered to the position closest to the magnet.

Using the SFL process, the hydrogel achiral crescent microswimmers were fabricated (the detailed fabrication process is described in the Methods Section). The SEM image (Figure 2A) shows the porous morphology of a hydrogel microswimmer, which was caused by dehydration of the sample. The dispersion of Fe_3_O_4_ can influence the fabrication process of the hydrogel microswimmers as a non-uniform dispersion can lead to uneven polymerization of the hydrogel precursor suspension. As shown in Figure 2B, EDS measurements show the distribution of Fe_3_O_4_ content in the hydrogel microswimmer; the result showed that the Fe_3_O_4_ nanoparticles were uniformly embedded inside the hydrogel microswimmer. The precursor suspension under the optical microscope also showed good dispersion (Figure 2C), which enables the uniform polymerization of the magnetic hydrogel suspension. To verify that the nanoparticles were magnetic, a neodymium iron boron magnet was placed close to the hydrogel precursor suspension to attract the nanoparticles; as expected, the magnetic nanoparticles inside were attracted to the sidewall of the container and quickly aggregated (Figure 2D).

### 3.2. Degradability of the Hydrogel Crescent Microswimmers

**Figure 3:**
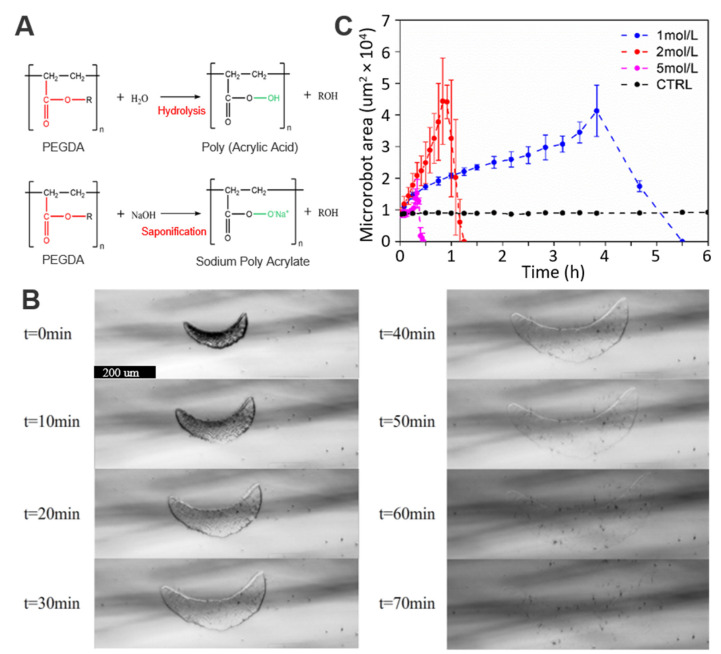
(**A**) First row: hydrolysis of the hydrogel microswimmer; second row: accelerated hydrolysis under the action of sodium hydroxide. (**B**) Optical microscope images of the degradation process of a hydrogel microswimmer with a 2 mol/L NaOH solution; the sample size was 3. (**C**) Relationship between the degradation time of the microswimmers and the concentration of the NaOH solution.

The degradability of the PEGDA hydrogel crescent microswimmers was quantitatively studied. The degradation properties are derived from the hydrolysis of esters of PEGDA. Water molecules break the ester groups in PEGDA (Figure 3A), resulting in the formation of poly (acrylic acid) (PAA) and alcohol moieties (PEG or PE), all of which have low cytotoxicity and can be directly excreted by the human body. Moreover, hydrolysis is accelerated by NaOH due to the presence of hydroxyl ions. When the hydrogel microswimmers are placed in NaOH solution, NaOH cleaves the ester group, causing the PEGDA polymer matrix to gradually break and disintegrate. Figure 3B shows the degradation process of a representative PEGDA hydrogel microswimmer when exposed to a NaOH solution with a concentration of 2 mol/L. First, the hydrogel microswimmer expanded. The expansion phenomenon happens because the PEGDA polymer matrix is broken down by NaOH, allowing water molecules to penetrate the hydrogel. When the strength of the PEGDA polymer matrix was greatly reduced and the original structure could not be maintained, the PEGDA polymer matrix gradually collapsed. Finally, the volume gradually decreased, and the microswimmer disappeared completely after 70 min. The degradation process of the hydrogel microswimmer was observed under different concentrations of NaOH solution (1 mol/L, 2 mol/L, and 5 mol/L), as shown in Figure 3C. The degradation rate of the microswimmer increased with the concentration of the NaOH solution. It should be noted that the composition of hydrogel materials can be changed to adjust the degradation time [26]. This is potentially useful because faster degradation may be required to achieve faster drug accumulation rates in actively controlled drug release applications [6]. In addition, the residual Fe_3_O_4_ nanoparticles after the full degradation of the hydrogel microswimmers are biocompatible and can be excreted by the human body [27].

### 3.3. Biocompatibility of the Hydrogel Crescent Microswimmers

**Figure 4:**
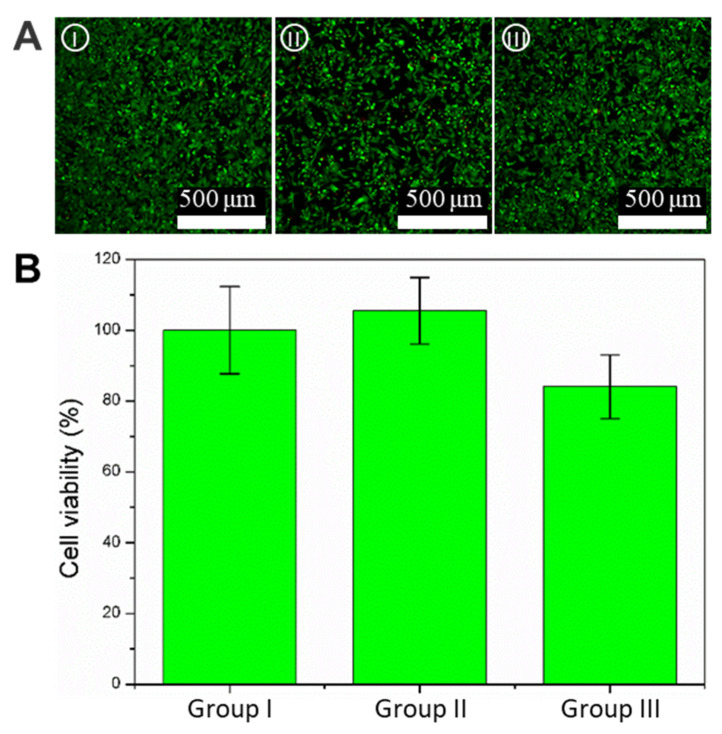
Biocompatibility test of the hydrogel crescent microswimmers. (**A**) Representative image of live/dead cell staining. Live cells (green) were stained with Calcein-AM, and dead cells (red) were stained with Calcein-PI. Groups I–III are the control group, the pure hydrogel group (microswimmers without magnetic nanoparticles), and the magnetic hydrogel microswimmers (microswimmers with 5 mg/mL magnetic nanoparticles), respectively. The scale bar is 200 μm. (**B**) Cell viability after 24 h incubation for groups I–III. The sample size was 3.

The biocompatibility of fabrication materials is an important consideration in the fabrication of microswimmers for microscale applications; thus, the biocompatibility of the hydrogel microswimmers was tested and compared using three groups of experiments: the control group (petri dish), pure hydrogel microswimmers (microswimmers without magnetic nanoparticles), and magnetic hydrogel microswimmers (microswimmers with 5 mg/mL Fe_3_O_4_); they will be designated as Group I, Group II, and Group III. After 24 h of incubation, the L929 cell viability of each group was evaluated by the calcein-AM/PI staining and CCK-8 kit. The fluorescence images enabled the visualization of live (green) and dead (red) cells in Figure 4A, which shows cell growth across the three groups well after 24 h. Almost no red fluorescence can be seen in any of the groups, indicating no discernible cytotoxic effect on cells. In addition, a CCK8 assay was used for the quantitative determination of cellular viability. The cell survival rates for the three groups were 100 ± 12.3%, 108.7 ± 8.8%, and 87.3 ± 8.1%, respectively, as shown in Figure 4B. Group II showed a survival rate greater than 100%; this means the hydrogel promoted cell growth, verifying the excellent biocompatibility of PEGDA. The survival rate from Group III was less than that of Group I due to the magnetic nanoparticles being slightly toxic to cells [27]. In general, hydrogel crescent microswimmers have good biocompatibility and ensure the safety of the biological tissue during a task.

### 3.4. Magnetic Actuation of the Hydrogel Microswimmers

**Figure 5:**
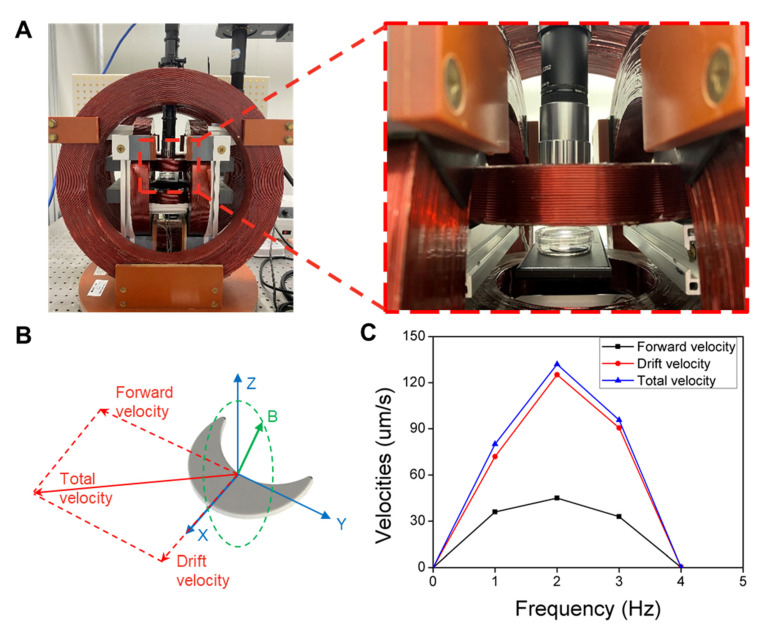
Biocompatibility test of the hydrogel crescent microswimmers. (**A**) Photograph of the magnetic control system. The zoomed-in image shows a sample of the microswimmers inside a petri dish placed at the central platform of the system. The position of the sample was adjusted to be as close as possible to the center of the coils to ensure field uniformity. (**B**) Schematic of a microswimmer and its swimming and drifting directions relative to the rotating magnetic field displayed in Cartesian coordinates. (**C**) The velocity profiles of the microswimmers. The step-out frequency is 2 Hz, and the maximum forward velocity is 42 μm/s.

In previous work, the swimming ability of crescent microswimmers has been proved theoretically and demonstrated experimentally [28,29,30]. To further quantify the swimming ability of the hydrogel crescent microswimmers on swimming performance, magnetic drive tests were performed. Here, a customized three-dimensional Helmholtz coil (Figure 5A) was used to generate a uniform RMF to drive the microswimmers. As shown in Figure 5B, the RMF acts on the microswimmers to generate a magnetic torque and makes the microswimmer rotate synchronously with the external magnetic field; this allows the microswimmers to convert rotational motion into translational motion and swim forward in a direction parallel to the axis of rotation. The RMF used for magnetic control can be expressed as [31]:

(1) $$B=\left[\begin{array}{c} B_{r}\sin\left(\theta \right)\cos\left(\omega t\right)\\ B_{r}\cos\left(\theta \right)\cos\left(\omega t\right)\\ B_{r}\sin\left(\omega t\right) \end{array}\right],$$

where $B_{r}$, θ, ω, and t represent the amplitude of the rotating magnetic field, the heading angle, the rotating frequency of the field, and time, respectively. Since the microswimmers were actuated close to the surface, the velocity was decomposed into two components: forward velocity and drift velocity [32]. Forward velocity is defined as the velocity measured along the direction of the rotational field normal while drift velocity is defined as the velocity perpendicular to it (Figure 5B). The drifting motion is due to the interaction between the microswimmer and the surface of the substrate. Figure 5C shows the velocity profiles of the hydrogel microswimmers as a function of the rotation frequency of the applied 10 mT RMF (the corresponding information is provided in video S1). From 1 Hz to 4 Hz, the forward velocity of the hydrogel microswimmers first rises and then falls. At 2 Hz, they have a maximum forward velocity of 42 μm/s. The corresponding dimensionless swimming efficiency is *V*_max_/(*Lf*) = 0.13 (*V*_max_ = 42 µm/s, body length *L* = 161 µm, frequency *f* = 2 Hz), which is on par with previously reported achiral microswimmers [28]. The low step-out frequency at 2 Hz can be explained by the low loading efficiency of Fe_3_O_4_ particles; the same issue had been observed with previously reported hydrogel microswimmers [6,12,33].

## 4. Conclusions

In this work, we demonstrated a new type of fabrication method for degradable hydrogel crescent microswimmers based on a custom-built SFL setup. The SFL setup enabled the fabrication of hydrogel crescent microswimmers with high throughput and low cost. The degradability experiments tested the degradation rate of the hydrogel microswimmers under different concentrations of a NaOH solution and demonstrated the complete degradation of the hydrogel crescent microswimmers. Cytotoxicity experiments showed that the hydrogel crescent microswimmers have good biocompatibility, ensuring their biosafety to the environment. A customized magnetic control system was used to test the swimming motion of the microswimmers. The swimming efficiency of the microswimmers was 0.13, which is on par with previously reported achiral microswimmers. The results showed that the hydrogel crescent microswimmers were a promising candidate for microscale applications due to their swimming efficiency and degradability. For future work, we will implement drug loading and controlled release functions on these hydrogel microswimmers to further demonstrate their potential for biomedical applications. Furthermore, we will consider using in situ synthesis technology to further improve the loading efficiency of Fe_3_O_4_ and magnetically align the magnetic nanoparticles during the fabrication process to enhance the magnetic property of the hydrogel microswimmers; these can potentially improve the swimming capability of the microswimmers by increasing the step-out frequency.

## Data Availability

All data generated or analyzed during the present study are included in this published article.

## Supplementary Materials

The following are available online at https://www.mdpi.com/article/10.3390/mi13050798/s1, Figure S1: The custom-built stop-flow lithography setup, Table S1: The crosslinking yield and Swelling efficiency of the PEGDA hydrogel.
