# Supplementary material for: Stop-Flow Lithography for the Continuous Production of Degradable Hydrogel Achiral Crescent Microswimmers
Source: Micromachines (Basel). 2022 May 20;13(5):798. doi: 10.3390/mi13050798 (PMC9144168; doi:10.3390/mi13050798)
Supplement: Supplementary file 1 [file micromachines-13-00798-s001.zip › micromachines-1719795-Supplementary.pdf]

micromachines

Supplementary Materials

Stop-Flow Lithography for the Continuous Production of Degradable Hydrogel Achiral Crescent Microswimmers

Junfeng Xiong, Xiaoxia Song, Yuhang Cai, Jiahe Liu, Yangyuan Li, Yaqiang Ji, Liang Guo and U Kei Cheang

1. Swelling property test of hydrogel

Result: The crosslinking yield of PEGDA is 33.5%, swelling efficiency is 100.3%

Method: After freeze-drying PEGDA hydrogel, a certain amount of hydrogel was weighed and marked M_0. The freeze-dried sample was soaked in deionized water, and the water was changed every 8h. After swelling at 25℃ for 36 h, the water on the surface of the hydrogel was absorbed with absorbent paper, weighed and marked as M_1. The hydrogel was then freeze-dried for 36 h, weighed and labeled M_2. The crosslinking efficiency of hydrogel is $(M_0- M_1)/ M_0*100\%$; Swelling rate yield $(M_2- M_1)/ M_1*100\%$, 3 parallel experiments were conducted, and the average was taken.

Table S1. The crosslinking yield and Swelling efficiency of the PEGDA hydrogel.

Group	M_0(g)	M_1(g)	M_2(g)	crosslinking yield = $(M_0- M_1)/M_0*100\%$	Swelling efficiency = $(M_2- M_1)/M_1*100\%$
1	1.58	0.52	1.059	32.91139	103.6538
2	1.5	0.469	0.782	31.26667	88.0597
3	1.7	0.617	1.290	36.29412	109.0762
Mean				33.491	100.263
Std				2.093	8.909

2. The custom-built stop-flow lithography setup

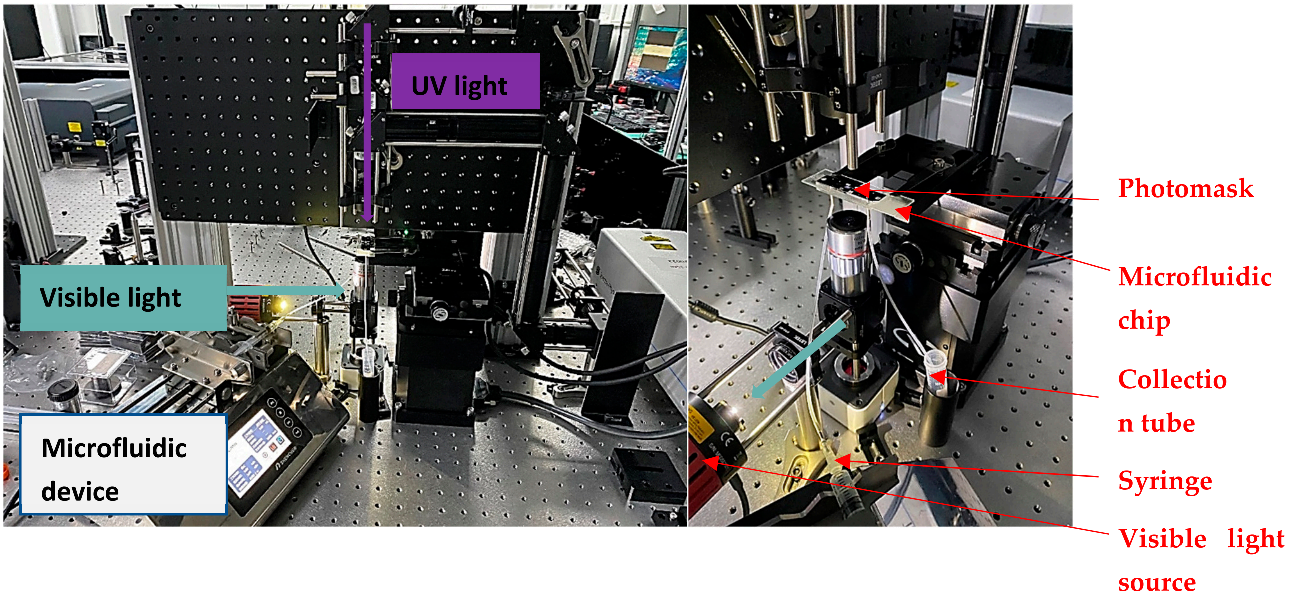

Figure S1. The custom-built stop-flow lithography setup.
